# From Beyond the Grave: Use of Medical Information from the Deceased to Guide Care of Living Relatives

**DOI:** 10.1007/s40142-020-00196-6

**Published:** 2020-11-24

**Authors:** Shereen Tadros, Helena Carley, Anneke Lucassen

**Affiliations:** 1 North East Thames Regional Genetics Service, Great Ormond Street Hospital, London, UK; 2 St. George’s University of London, Cranmer Terrace, Tooting, London, SW17 0RE UK; 3 South East Thames Regional Genetics Service, Guy’s Hospital, London, UK; 4 South West Thames Regional Genetics Service, St George’s Hospital, London, UK; 5 Faculty of Medicine, University of Southampton, Southampton, UK

**Keywords:** Next of kin, Access to records, Genetics, Consent, Confidentiality

## Abstract

**Purpose of Review:**

In order to inform patients of their genetic risks, access to the medical records and/or stored samples of their relatives is often helpful. We consider some of the obstacles to such access when these relatives are deceased and suggest how they might be navigated.

**Recent Findings:**

We explore an issue first highlighted in 2004 by Lucassen et al. (Br Med J 328:952–953, 2004) and re-evaluate it in the wake of novel technologies and mainstreaming of genomic medicine. We find that it is still an issue in practice despite professional guidelines advocating access to familial information (Joint Committee on Genomics in Medicine 2019) and that the Human Tissue Act 2004 is often wrongly constructed as a reason to block access. Access is often obstructed by failing to adopt the necessary relational concept of autonomy that applies in genetic medicine as reported by Horton and Lucassen (Curr Genet Med Rep 7:85–91, 2019) and by considering confidentiality to be absolute, even after death. In response to a recent legal case about the confidentiality of genetic test results, and their disclosure to family members (ABC v St George’s Healthcare NHS Trust 2020), Dove et al. (J Med Ethics 45:504–507, 2019) suggested that a duty to consider the interests of genetic relatives could co-exist alongside a duty of confidentiality to a patient. In this way, healthcare professionals can use professional judgement about the relative value of genetic information to family members. This is equally relevant in accessing deceased relatives’ information. A recent systematic review found a high level of acceptability of postmortem use of genetic data for medical research amongst participants and their relatives, and it is reasonable to assume that this acceptability would extend to clinical practice as reported by Bak et al. (Eur J Hum Genet 28:403–416, 2020).

**Summary:**

Within clinical practice, access to medical records/samples of deceased relatives is often obstructed unnecessarily, potentially resulting in harm to the living relatives seeking advice. Consent to such access is important but need not be the bureaucratic hurdle that is often imposed.

## Introduction

We are in a new era of genomics, in which genetic analysis is significantly cheaper and faster than it was even just a few years ago. However, despite the significant advances in molecular diagnostics, genetic medicine is still very often aided by, and at times dependent on, the simple step of reviewing a family history. In some cases, performing a careful scrutiny of relatives’ medical records for histological details of diseases they have had and testing a relative for particular genetic variants are prerequisites for accurate genetic testing in the person seeking advice.

Whilst clinical practices involving genetics and genomics are not alone in relying on a familial approach (the current pandemic has highlighted how the diagnosis of one patient brings other patients to the fore), the tensions raised are subtly different. In genetics, we need family members to provide evidence of something that happened, often many years previously, the inheritance—or not—of particular traits. The equivalent of a contact trace in infectious diseases might then be to find out which relatives need some form of surveillance or treatment to ameliorate the inherited risks they find themselves to be at.

Indeed, patients seeking genetic advice are often asked to obtain further information about the details of their relatives’ conditions even before they attend the genetics clinic so that their family history can help direct investigations. For living relatives, a key step in obtaining such information is their consent. Services employing family history approaches will have pathways to obtaining the consent from living relatives to access relevant details of their medical history. Information from deceased relatives might be equally valuable in this family history quest, but seeking their consent is clearly no longer possible and very few people leave clear, specific wishes about who can access their medical records posthumously. The reasons why such access might be necessary have not been part of public conversations in the same way that debates about, for example, organ donation have resulted in more explicit declarations before death. As a result, reticence on the part of record or tissue sample holders to release information is common. This reticence often results in requests—from the data holders—for some form of proxy consent, often from the “next of kin” of the deceased.

Access to stored samples from the deceased (for example, a tumour biopsy taken as part of their diagnostic workup) might be equally important in the search for inherited traits. Public awareness of routine storage of diagnostic samples in pathology archives, even beyond death, appears limited. Yet genetic testing on a stored sample of tissue/blood/DNA may be the most effective investigation to clarify inherited predispositions. The Human Tissue Act 2004 (HTA) [1], introduced in the wake of organ retention scandals, provides a legal framework for accessing samples from a deceased person but introduces different rules for samples that were taken before death (e.g., the tumour biopsy example above) and treats stored tissue differently from stored DNA samples. The underlying principle for access to samples under the Human Tissue Act is that of consent, but not necessarily from a “next of kin.”

As a result, considerable confusion has arisen in routine NHS practice about what permissions are required for access to data or samples in the practice of genetic medicine. In this paper, we discuss the ethical, legal, and practical issues around access to medical records or stored samples of a deceased person and suggest practical ways forward. We start with a fictionalised scenario of what happens in practice.

## Fictionalised Scenario

Ms. A contacts her sister (Ms. B) to inform her that she has been diagnosed with terminal bowel cancer. Ms. A’s doctors have told her she may have a familial predisposition to developing cancer and have referred her to her local clinical genetic service but she dies before she is able to attend the appointment.

Ms. B is now referred to her local clinical genetic service to find out about her own risk of cancer, that for her children, and what screening or risk-reducing measures might be available. In order to inform Ms. B, the clinical genetics team would like the following:

- Access to Ms. A’s medical records to confirm her diagnosis (different cancers are associated with different familial tendencies)
- Possible access to Ms. A’s stored tumour tissue for testing

The histopathology department asks for consent from Ms. A’s next of kin before they will release the report or samples. Mr. C (Ms. A’s spouse) is approached via clinical genetics but does not respond to requests for consent to access either Ms. A’s medical information or tests of the tumour stored in the pathology department as part of Ms. A’s medical record.

How should the clinical genetics team proceed? Is consent required firstly for release of Ms. A’s medical records and secondly for testing of her tumour tissue? If so, who may provide such consent? Should Mr. C have been approached at all?

## What or Who Is Next of Kin?

The term “next of kin” (NoK) is commonly used to refer to a person’s closest living relative, but it is in fact a lay term and it is not recognised in UK law. However, the question “who is your next of kin?” is often asked within healthcare, for example, on admission to a hospital or on registering with a GP. The NoK can be whomever that person would like; it could be a relative but could also be a close friend.

The reason for appointing a NoK is so that when a patient is incapacitated, the NoK can help inform what the patient might have wanted in that particular situation or give an appreciation of a patient’s prior circumstances. The role of the NoK in this setting is someone who can represent the patient’s interests if they are not able to do so themselves, but importantly, they are not afforded the ability to consent on behalf of the patient and they have no legal right to make or refuse any treatment decisions on the patient’s behalf [2].

After death, the HTA governs the removal, storage, or use of cadaveric material and determines which relative can consent to which activity. The HTA does not utilise the term NoK but does use the term “qualifying relationships” in relation to those from whom consent must be obtained.

However, for samples taken before death (but where the sample donor has subsequently died), the patient’s consent is presumed to have been taken for removal and storage, and thus, they are not governed by the HTA. Further testing on these stored samples after the patient has died will not require any new consent if the testing is to refine a diagnosis in the (now) deceased person. However, the main reason for such refinement is often for the benefit of a relative (for example, does immunohistochemistry of a tumour indicate a heritable component), and it is then a moot point whether further consent is required.

It is worth noting here that the General Data Protection Regulation (GDPR) which came into force by the Data Protection Act 2018 [3], whilst giving automatic access to a person’s own records, does not apply when trying to access the records of a deceased relative.

The question we are left with, then, is this: would the histopathology department be breaching Ms. A’s confidence in divulging information without consent from someone? And if so, who should that someone be?

At this point, it is worth considering the possible arguments against such disclosure.

## Confidentiality in Death

A patient’s right to confidentiality has been a tenet of medical ethics since Hippocratic times [4] and is generally considered to continue after death. The World Medical Association Declaration of Geneva, for example, says, “I will respect the secrets which are confided in me, even after the patient has died” [5]. The British Medical Association [6] and the General Medical Council (GMC) also consider that the “duty of confidentiality continues after a patient has died” [7]. Does this duty mean that a deceased patient’s medical information should never be released, and does the context of such a release matter?

### Interests of the Dead: Can the Dead Be Harmed?

Whilst a dead body does not have interests, a person’s interests whilst alive should and do continue after death. This is why we make wills and abide by them. There are moral duties incumbent on the living to carry out the will of the deceased, but does this extend to not allowing access to information that might guide the treatment of relatives? This situation is not envisaged in most of the guidance about confidentiality after death. The GMC notes that disclosure of medical information after death requires a balancing exercise that includes the following:

whether and what personal information may be disclosed after a patient’s death will depend on the facts of the case. If the patient had asked for information to remain confidential, you should usually abide by their wishes. If you are unaware of any instructions from the patient, when you are considering requests for information you should take into account:

- Whether the disclosure of information may cause distress to, or be of benefit to, the patient’s partner or family;
- Whether the disclosure of information about the patient will, in effect disclose information about the patient’s family or other people;
- Whether the information is already public knowledge or can be anonymised;
- The purpose of the disclosure. [8]

Where a deceased patient has specifically said that they do not wish certain information to be disclosed, then this fact should be considered in the balancing exercise, but a blanket no-disclosure policy without NoK consent does not do this justice. Whilst it may well be helpful to gather information about the wishes of the deceased, sensitivity must be employed: the very act of approaching a bereaved person to request consent may be viewed as insensitive at best and harassment at worst.

In our example, Ms. B was informed by Ms. A of her diagnosis, suggesting that this was not a secret she wished to keep private. The lack of specific consent for Ms. B to access this information means little, since many people will not understand the importance of their specific information in the genetic diagnosis of relatives.

Having considered the potential for posthumous harm, it is pertinent to consider whether posthumous benefits might also arise. The concept of posthumous redemption is often the motivation behind organ donation: making sense of the loss of a loved one and allowing them to “live on” beyond death [9]. One could consider that the good that can be done by allowing family members to understand their level of genetic risk has a similar redemptive quality.

### Interests of the Living

The interests of current patients also need to be considered. They might reasonably expect their medical records to be kept secret after death and might be reticent to disclose important information should they feel this seal is easily broken. However, this approach misunderstands the nature of the disclosure. The clinical genetic enquiry in our case is about the detail of already shared information to help direct testing in relatives. This is not a request for Ms. B to access the secrets of her relatives but an NHS service utilising its resources appropriately by using family history information to target its search. Genetic medicine has the potential to influence a range of relatives in different ways and in ways that cannot be easily captured by considering the confidentiality of, or consent from, any particular person.

In our case, Mr. C was approached for consent, but given he was Ms. A’s spouse and not her blood relative, the resultant information is not for his medical benefit. It could indeed be that his lack of engagement reflects the fact that he does not understand the reasons behind the request, and to explain these would arguably be to breach the confidence of Ms. B. We have previously argued that seeking consent in this way is misguided [10], but UK cancer genetic services are still frequently denied access unless there is consent from “the NoK” [11].

Failure to release medical information relating to Ms. A has the potential to cause harm in denying Ms. B—and other relatives—timely access to knowledge about an inherited predisposition, which would in turn enable access to relevant surveillance and prophylaxis, meaning that cancer diagnoses may be missed or made at a stage where the prognosis is worse. Equally, lack of access to this information could mean that decisions are made on the basis of an incorrect tumour type, resulting in unnecessary surveillance. This has its own inherent harms, not only in terms of the potential for unnecessary anxiety but also depending on the type of screening, which may lead to direct physical harms, including colonoscopic perforation [12] or unnecessary surgery [13]. Further, unnecessary surveillance puts unjustified pressure on a healthcare system already under strain, and so it could be argued that it is in the wider interests of society to disclose information which could ease this burden.

### Does Access to Ms. A’s Records Engage Rules on Confidentiality in This Setting?

We suggest that the reticence around releasing medical information of the deceased stems from a well-intentioned misunderstanding of the concept and context of medical disclosure. The very term “disclosure” often has a negative connotation, with the Cambridge English Dictionary specifically pairing it with the word “damaging” in both its context examples [14].

In the context of clinical genetics, disclosure is not the act of making secret information publicly available. The information sought in such cases is generally a matter of confirming details or gaining a fuller picture of an already established and acknowledged diagnosis, by health professionals who are bound by their professional codes of practice to only utilise that which is necessary. The medical records are not “shared” more widely, and often the only information that needs to be divulged to relatives is that the information has helped clinicians narrow down the likely familial tendency.

It is also worth noting that rules around data sharing between trusts [15] are often misinterpreted. The Access to Health Records Act 1990 states that where a patient has died, their medical records may be released to their personal representative or to someone who has a claim arising from the death [16].

However, the Act also states that trusts can withhold that information if the deceased patient had an expectation that it would not be disclosed. As the general duty of confidentiality is well established in public understanding, it would be a rare patient who would *expect* disclosure of their medical information, outside of the clinical genetics setting. Even if they did expressly consent for it, such consent would need to be documented in an easily searchable way, as is demographic information. This caveat, taken to its extreme, would mean that we must never disclose any information about the deceased in the absence of express consent in life.

Given that a death certificate is a public document, and information about cancer diagnoses is collected by the NHS’s cancer registry without asking for a patient’s consent, genetic services can access information about a deceased patient without consent from a relative or NoK. So, Ms. A’s diagnosis could be retrieved from her death certificate or cancer registry without Mr. C or anyone else’s consent, but access to Ms. A’s tumour for genetic testing cannot be done by either of these routes.

## Testing of Stored Samples After Death

Ms. A’s cancer has been confirmed, and the family history suggests the possibility of Lynch syndrome, an inherited genetic condition predominantly predisposing to bowel, endometrial, and ovarian cancers, but there are no affected living relatives in whom to initiate genetic testing. Immunohistochemistry of Ms. A’s stored tumour sample and testing for microsatellite instability would be the next step in advising Ms. B, with possible germline/somatic DNA analysis to identify the responsible genetic variant. The diagnostic testing may then allow predictive genetic testing of Ms. B—and other living relatives—to see whether or not she has inherited this familial tendency.

Why not simply test Ms. B for such a familial tendency? Such testing is much more accurate if the familial cause is known, and testing can be targeted for a specific familial variant—false negatives and false positives are much more likely without the supporting evidence from a family member with the condition in question.

### How Does the Human Tissue Act Affect Testing?

Public enquiry following several organ retention scandals illustrated how the retention of children’s organs was viewed as a kind of posthumous harm, for example, affecting the way a parent was able to grieve and remember their child [17]. This and other examples resulted in the reform of previous tissue legislation dating from 1961 to the Human Tissue Act 2004 enacted in 2006.

This Act provides a legal framework governing the way in which human tissue samples may be used, stored, and, in the case of the deceased, removed. Tissue samples are considered “relevant materials” and include material which “consists of or includes human cells” [18]. Several exclusions apply, such as gametes, embryos outside the human body, hair, and nail from a living person, cell lines, and, notably, extracted nucleic acid (DNA) [19]. With “appropriate consent,” the HTA permits the use of samples in “obtaining scientific or medical information about a living or deceased person which may be relevant to any other person (including a future person)” [20].

Whilst consent is the main route to accessing tissue, how this consent is given and by whom is complex [21, 22]. After death, consent may be evident from the wishes of the deceased individual immediately before she died, from her “nominated representative,” or from an individual who had a “qualifying relationship” with the deceased [23]. For the analysis of cellular material, consent must be provided by someone in a “qualifying relationship.” Although the term NoK is not used, individuals who may provide consent are ranked so that those with the highest ranking have precedence:

(a) spouse, civil partner or partner;

(b) parent or child;

(c) brother or sister;

(d) grandparent or grandchild;

(e) child of a person falling within paragraph (c);

(f) stepfather or stepmother;

(g) half-brother or half-sister;

(h) friend of longstanding. [24]

The Act recognised the possibility of testing for familial reasons, and as a result, the analysis of DNA held in cellular material can be authorised by any individual on the list [25]. Indirect assessment of DNA (such as immunohistochemistry) can then also be authorised by anyone on the list in this same spirit. It is worth noting that the HTA only covers cellular material so that stored DNA samples (the bread and butter of genetic and genomic services) do not fall under the Act. These are instead subject to common law and professional guidance [26•]. Importantly, the ranked list is for samples taken post mortem. Where—as in Ms. A’s case—the sample was taken ante mortem but the patient has since died, consent for testing should not need to adhere to a ranked list (the rationale being that in this case, biological relatives of the deceased person have a higher stake in establishing a genetic diagnosis than the person’s spouse).

This means that asking Mr. C’s consent for access to the medical records or stored tissue of Ms. A was not necessary. We note that many NHS services are not aware of, for example, the complexities around the HTA and thus create hurdles for access that are not present in professional guidelines or legislation.

## Could an Alternate Approach Replace the Requirement for Formal Consent to Access Medical Information from the Deceased?

We have seen how a NoK is primarily someone who can inform health professionals what an incapacitated patient might have wanted whilst capacious. Does and should this role extend to protecting the interests of the dead? We argue that in the case of familial investigations in genetic medicine, this makes no sense. Asking Mr. C for the release of information on his spouse, for the benefit of someone he is not biologically related to, has no basis in law or professional guidelines. If the investigation yielded familial information, then it is true that others might need to be informed, for example, Ms. A’s children, and this might best be done via Mr. C, but this we argue is a different issue.

We propose an alternate approach. We consider that access to familial information by genetic services should be viewed in the same way as cancer registration—it is in the public interest to collect such information, and access by NHS genetic services should be permitted on the basis of facilitating familial investigations in order to provide accurate risk assessments [27]. Clinical genetic services would be well placed to act as intermediaries to handle familial information from the deceased where this may benefit the living. It should be possible to facilitate such investigations whilst maintaining the confidentiality of the deceased. After all, details can only be sought if a family member already knows their relative had cancer.

If pathology departments continue to view consent as the key to unlock the safe of medical information, then it is clear that the consent of a close relative with a legitimate interest, such as Ms. B, should suffice. (In fact, as a blood relative, we consider Ms. B’s consent as arguably superior to that of Mr. C.) Does such consent need to be a signed document? It may be that pathology departments require a paper trail confirming that consent has been obtained, but we believe that it should be sufficient for the clinician to state that she has the consent of the relative. Given the current and ongoing challenges of telemedicine, this approach would likely be far more efficient.

## Is Information That Is Potentially Familial Confidential?

Whilst a particular diagnosis or condition can clearly be said to be something that should be kept confidential, the question of whether the familial factor that predisposed to the condition is confidential in the same way has long been questioned. If a particular genetic variant has been inherited by several family members, why should that be confidential to the person in whom it was first detected? Thus, giving the index patient a right of veto over its discovery in relatives would seem inappropriate [28, 29].

## Conclusion

Lucassen et al. [10] made the case in 2004 that consent should not be a prerequisite in order for pathology departments to release medical information to clinical genetic departments about deceased relatives. In our experience, this continues to be a problem, and misinterpretation of the HTA has further complicated this issue.

We argue that the use of medical information from deceased relatives for clinical genetic enquiries is not disclosure in the generally understood sense of the word and should not be viewed in the same way as a breach of confidentiality.

Requests from clinical genetic services are made in response to referrals of at-risk relatives, and these relatives provide consent to genetic services to enquire on their behalf about inherited conditions. With this in mind, we suggest that formal consent for access to medical information of deceased patients is not necessary and should not be sought from the NoK. Instead, such disclosures should be permissible as an essential feature of genetic medicine services. Where necessary, verbal consent, taken by the clinical genetics team, should suffice. We would welcome such a pragmatic approach which we believe is overdue and would greatly assist in the provision of care to patients seeking genetic advice.
